# Current progress and critical challenges to overcome in the bioinformatics of mass spectrometry-based metaproteomics

**DOI:** 10.1016/j.csbj.2023.01.015

**Published:** 2023-01-16

**Authors:** Nobuaki Miura, Shujiro Okuda

**Affiliations:** a Division of Bioinformatics, Niigata University Graduate School of Medical and Dental Sciences, 2-5274 Gakkocho-dori, Chuo-ku, Niigata 951-8514, Japan; b Medical AI Center, Niigata University School of Medicine, 2-5274 Gakkocho-dori, Chuo-ku, Niigata 951-8514, Japan

**Keywords:** Metaproteomics, Mass spectrometry, Gut microbiome, Bioinformatics, Amino acid sequence database, False discovery rate

## Abstract

Metaproteomics is a relatively young field that has only been studied for approximately 15 years. Nevertheless, it has the potential to play a key role in disease research by elucidating the mechanisms of communication between the human host and the microbiome. Although it has been useful in developing an understanding of various diseases, its analytical strategies remain limited to the extended application of proteomics. The sequence databases in metaproteomics must be large because of the presence of thousands of species in a typical sample, which causes problems unique to large databases. In this review, we demonstrate the usefulness of metaproteomics in disease research through examples from several studies. Additionally, we discuss the challenges of applying metaproteomics to conventional proteomics analysis methods and introduce studies that may provide clues to the solutions. We also discuss the need for a standard false discovery rate control method for metaproteomics to replace common target-decoy search approaches in proteomics and a method to ensure the reliability of peptide spectrum match.

## Graphical Abstract

**Figure:**
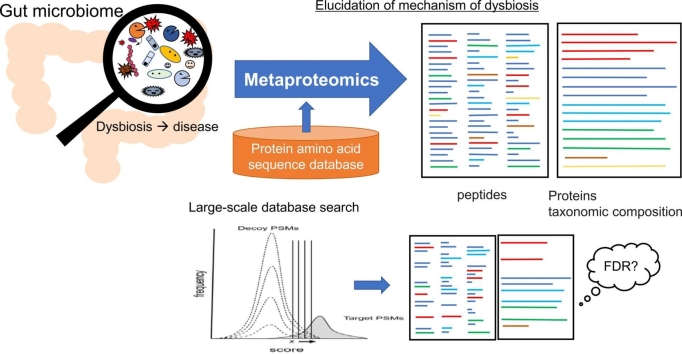

## 1 Introduction

Metaproteomics using mass spectrometry (MS) is a powerful technique that profiles proteins expressed by all of the organisms present in extremely diverse biological and ecological samples. The term metaproteome was first used by Wilmes and Bond in 2004 to refer to “the dynamic functions of a mixture of microorganisms at a given point in time [1]”. Metagenomics is a static catalog of the genes present across the species of a sample, while metaproteomics provides a dynamic catalog of species and expressed proteins in terms of which genes are expressed and which species possess which function. Metaproteomics of a wide variety of complex microbial and ecological systems, including gut bacteria [2], [3], [4], [5], [6], saliva [7], oral environment [8], ocean water [9], hydrothermal vents [10], plant lignin degradation [11], soil rhizosphere [12], and brewing vinegar [13], have been studied to obtain inter/intra communication/networks *via* cataloging of proteins and species. The collected information is invaluable for understanding the mechanisms of biological systems, including homeostasis, disease, and differentiation [14].

Using PubMed (https://pubmed.ncbi.nlm.nih.gov), we searched for "proteomics" and "metaproteomics" to determine the number of published articles. The results show that the number of articles on both proteomics and metaproteomics is increasing annually. However, in 2021, 16,175 articles on proteomics had been published, while only 145 on metaproteomics had been published. The first article on the intestinal microbiome was published in 2009 [15], and it has been 13 years since metaproteomics of the intestinal microbiome began. By this preliminary survey, the number of articles on metaproteomics is 1/100 of those on proteomics, even though metaproteomics is an extension of proteomics. Metaproteomics is still in its infancy, and several challenges must be overcome before it can be used for routine research. These issues are as follows:

1) Issues in database construction: it is difficult to construct an appropriate amino acid sequence database for mass spectrometry analysis because of the diversity of microbiota.
2) Issues associated with false identification in the database search: problems arise in searches in a large database.
3) Issues in spectrum acquisition issue: it is necessary to devise a method for spectra acquisition since many spectra are obtained in metaproteomics.

In this review, we introduce recent research on metaproteomics of the human microbiome to demonstrate its usefulness and discuss the issues that remain to be solved.

## 2 Metaproteomics of human microbiome and disease

Microorganisms such as fungi, bacteria, and viruses form complex community called the microbiome. These microorganisms live in the human gut, skin surface, oral cavity, esophagus, and lungs. The number of cells in an average human adult body is approximately 30 trillion, whereas the number of symbiotic bacteria, including intestinal bacteria, is 39 trillion with over 1000 species [16]. Therefore, it can be said that half of all “human” cells are in fact symbiotic bacteria. Most symbiotic bacteria in humans are intestinal. Although other microbiota such as those in the oral cavity and skin are thought to be present in quantities that are more than one order of magnitude smaller, a vast number are still involved in biological processes. While humans provide nutrition and a suitable environment for microorganisms, microorganisms play essential roles in maintaining homeostasis, including defense against harmful pathogens [2]. An imbalance of the distribution of microorganisms in the microbiome and an abnormal physiological state is called dysbiosis. In particular, dysbiosis of the intestinal microbiome causes a variety of diseases, and considerable research on the involvement of intestinal bacteria in disease has been conducted [17], [18]. Dysbiosis of intestinal bacteria results in an increase in the number of harmful species. Toxic substances such as putrefactive products, bacterial toxins, and carcinogens produced by these bacteria directly damage the intestinal tract and are absorbed into the body through the intestinal epithelial space, where the barrier function is impaired. This is believed to cause damage to various tissues, such as the liver, heart, kidneys, pancreas, and blood vessels. Recently, it was shown that *Porphyromonas gingivalis*, which causes periodontal disease, may also cause dysbiosis of intestinal bacteria [19], [20]. This can be considered a type of bacterial-host interaction. Therefore, an analysis of the state of the intestinal microbiome presents a means to elucidate the disease mechanism. Using 16S rRNA gene analysis, it is possible to estimate the species and approximate abundance ratios of the intestinal microbiome. Therefore, metagenomic analysis can help reveal the entire complement of species and genes present in the intestinal microbiome. However, information on species and genes obtained from metagenomic analysis and 16S rRNA gene analysis is static. To determine which genes of which species are actually expressed and cause disease, it is necessary to analyze proteins. Metaproteomics of fecal samples can help elucidate the bacterial species and proteins associated with dysbiosis and thus understand the mechanism of the condition. Recent metaproteomic studies of several diseases are presented below.

### 2.1 Inflammatory bowel disease (IBD）

Inflammatory bowel disease (IBD), characterized by inflammation of the intestinal mucosa, is estimated to affect more than 0.3 % of the world population, and the number of children with IBD is increasing [21], [22]. Childhood-onset IBD differs from adult IBD in its progression, anatomical location, and treatment outcome. Childhood-onset IBD often results in growth retardation and serious long-term health consequences [23]. Therefore, there is an urgent need to better understand the pathogenesis of childhood-onset IBD.

Zhang et al. performed a large intestinal metaproteomic study of a pediatric inflammatory bowel disease (IBD) inception cohort [24]. Although intestinal bacteria are thought to be involved in the pathogenesis of IBD, the factors mediating the interaction between the host and microbiota are not known. The authors collected mucosal-luminal interface (MLI) samples from 71 untreated pediatric IBD patients (25 with Crohn’s disease (CD), 22 with ulcerative colitis, and 24 controls with non-IBD), and evaluated both gut bacteria and related human proteins using metaproteomics. The expression of microbial proteins related to oxidative stress responses was higher in IBD cases than in non-IBD cases. In particular, the expression of human proteins related to oxidative antibacterial activity was increased in IBD cases and correlated with changes in microbial function. For the first time, microbial functions and metabolic pathways were found to respond to oxidative stress, such as DNA damage repair, protection, mobilization, and L-cysteine degradation. They also found that *Faecalibacterium prausnitzii*, an intestinal commensal bacterium, was associated with CD at the strain level. Furthermore, they showed for the first time that host defense proteins, including proteins that produce reactive oxidants, are present in intestinal extracellular vesicles and correlate with functional changes in the microbiota in pediatric IBD.

### 2.2 Type 1 diabetes

Type 1 diabetes, caused by T cell-mediated destruction of insulin-producing cells, occurs under the influence of both genetic susceptibility and environmental factors [25]. Environmental factors, including infant diet and exposure to microbial agents, have been associated with the risk of type 1 diabetes, as the composition of the gut microbiota is shaped by both genetics and environmental factors such as diet and hygiene [26], [27], [28]. It has been hypothesized that dysbiosis of the gut microbiota exacerbates intestinal inflammation before disease onset [25]. Understanding the functional properties of the microbiota and the interactions between the gut microbiota and the host are necessary to dissect the role of the gut microbiota in the development of type 1 diabetes.

To test the hypothesis that intestinal inflammation is associated with the progression of type 1 diabetes and to characterize changes in the functional properties of the microbiota in individuals with islet autoimmunity and clinical disease, Gavin et al. performed a metaproteomic study of soluble fecal proteins in stool samples from new-onset patients (n = 33), islet autoantibody-positive subjects (n = 17), low-risk autoantibody-negative subjects (n = 29), and healthy subjects (n = 22) [29]. A total of 470 human proteins and 10,908 non-human protein clusters were identified. The average number of identified protein clusters per sample was 1566 ± 305. From the obtained proteins, we found 24 proteins that varied significantly by one-way analysis of variance, seven of which (galectin-3, fibrillin-1, CUZD1, neutral ceramidase, CELA3A, CLCA1, and IGHA1) were significant after multiple testing adjustments. Galectin-3 and fibrillin-1 were increased in new-onset patients, while CUZD1, neutral ceramidase, CELA3A, CLCA1, and IGHA1 were decreased in new-onset, islet autoantibody-positive, and islet autoantibody-positive patients. This is the first evidence that exocrine enzyme levels are reduced in islet autoantibody-positive subjects. Galectin-3 and fibrillin-1 levels are increased in new-onset patients, leading to the hypothesis that inflammation-related proteins are released into the stool in type 1 diabetes.

### 2.3 Ventilator-associated pneumonia (VAP)

Ventilator-associated pneumonia (VAP), which causes high morbidity and mortality among common nosocomial infections, is the second-most common nosocomial infection (HAI) in intensive care units (ICUs) [30] and its diagnosis is based on bronchoalveolar lavage (BAL) [31], [32]. However, BAL collection is invasive, making alternatives desirable. Pathak et al. collected BAL and endotracheal aspirates (ETA) from 16 intubated patients (11 with VAP, 5 controls) for 15 days and performed a metaproteomics analysis [33]. Although the abundance of microbial peptides was lower than that of host peptides and could not be quantified, 66 VAP pathogenic peptides were identified. Sixty-two of these peptides were detected in ETA, whereas only eight peptides were detected in the control. In contrast, only 10 peptides were detected in BAL. Among the VAP pathogen peptides in ETA, those derived from gram-negative bacteria were detected on multiple days, starting 1 day before the onset of VAP. These results suggest that ETA may aid in the early diagnosis of pneumonia in intubated patients.

### 2.4 Cystic fibrosis (CF)

Cystic fibrosis (CF) is a persistent lung infection that requires repeated antibiotic treatment [34]. Additionally, it affects pancreatic and intestinal functions, and the thickening of mucus in the small intestine reduces the absorption of nutrients and bile acids, which greatly affects the composition of the intestinal microbiome and causes intestinal inflammation [35]. In patients with CF suffering from dysbiosis, it has been noted that the abundance and diversity of enterobacteria of the phylum Firmicutes, especially butyrate-producing bacteria of Clostridium clusters IV and XIVa, are decreased [36]. Furthermore, metagenomic phylogenetic analysis has shown that *E. coli* is more abundant in patients with CF, suggesting that it contributes to gastrointestinal (GI) tract disease in CF [37]. Debyser et al. performed fecal metaproteomics of fecal samples from 15 children with CF and their unaffected siblings to obtain insight into the protein composition of hosts and microbes in the GI tract [38].

A total of 265,543 MS/MS spectra were obtained for 15 samples obtained from patients with CF and their corresponding unaffected siblings. In total, 98,321 peptides were identified (37 % identification rate) and assigned to 1676 proteins. Several inflammatory proteins such as Lipocalin-2 (NGAL), zinc-alpha2 glycoprotein, and orsomucoid1, were found to be greatly increased in the patient samples. Among these proteins, NGAL was stable. Taxonomic analysis of identified peptides showed that four main phyla (Firmicutes, Bacteroidetes, Actinobacteria, and Proteobacteria) that were commonly detected in the fecal microbiota of healthy individuals were also dominant in our data. The patient samples contained fewer Firmicutes and Actinobacteria but more Proteobacteria and Bacteroidetes than the sibling group. The observation of fewer Firmicutes in the patient samples at the genus level relies on the strong reduction in *Eubacterium*, *Roseburia*, and *Ruminococcus*. However, in the patient samples, the genera *Blautia* and *Clostridium*, which also belong to the phylum Firmicutes, increased, suggesting that these organisms may be markers of dysbiosis in CF patients.

### 2.5 Human immunodeficiency virus (HIV)

Human immunodeficiency virus (HIV) is a chronic inflammatory disease presenting with functional defects of multiple tissues such as enterocyte apoptosis, loss of epithelial integrity, cell-level defects such as errors in the production of mucin/IgA, and immunological defects such as loss of sub-mucosal lymphocytes, which contribute to an increased risk of mortality [39], [40], [41]. HIV infection induces dysbiosis of the gut microbiota, which correlates with immune status by enriching inflammatory and pathogenic bacterial populations and genes. The bacterial composition in this is differs between patients undergoing highly active antiretroviral therapy (ART) and untreated patients [42]. Serrano-Villar et al. examined protein synthesis in gut bacteria and accumulation of metabolites in the blood of 29 HIV-infected patients (nine untreated HIV-infected, 12 immunological ART responders, and nine non-responders) and eight healthy subjects [43]. HIV infection dramatically alters the activity of gut bacteria, which was more pronounced in ART responders. In previous studies, 16S rRNA gene analysis [40] and metagenome sequencing [42] at the genome level revealed that bacteria belonging to the Prevotellaceae, Bacteroidaceae, Desulfovibrionaceae, Enterobacteriaceae, Erysipelotrichaceae, Eubacteriaceae, Lachnospiraceae, and Veillonellaceae families have been reported as active biomarkers of HIV infection. In a study by Serrano-Villar et al., only the enrichment of proteins of the Acidaminococcaceae family was found to be an active biomarker for HIV patients by taxonomic profiling of expressed proteins. These results demonstrate the superior precision of metaproteomics in establishing the active mediators of disease pathology.

### 2.6 Leukemia patients with multidrug-resistant Enterobacteriaceae (MRE)

Infections caused by multidrug-resistant Enterobacteriaceae (MRE) during hospitalization are serious, especially in leukemia patients, who are often exposed to pathogens during long-term hospitalization owing to their weakened immune system. Additionally, antibiotic treatment causes changes in the microbiome [44]. Therefore, a better understanding of how the gut microbiota prevents colony formation by pathogens, and how such information can be used in the clinical management of patients, is important for preventing secondary infections. Rechenberger et al. performed a large-scale clinical metaproteomic analysis of 212 fecal samples from 56 patients with acute leukemia in whom MRE were established in the gut [4]. Seventy-two classes, 383 genera, and 595 species were identified in patient samples. The sample-specific taxonomic distribution of the proteome data and data from 16S rRNA gene analysis showed an average Pearson correlation of 0.95 at the class level (order: 0.94, family: 0.78, genus: 0.87). Taken together, these results suggest that it is possible to derive taxonomic distributions from metaproteomic analyses. The most abundant classes in all samples were Bacteroidia, Clostridia, and Verrucomicrobia, which is consistent with the results of previous studies [45], [46].

The most interesting point of this study beyond the large-scale metaproteomics, was the analysis of mass spectrometry data using various databases, highlighting the challenges of current metaproteomics based on mass spectrometry and database search. We discuss these challenges in detail in the following section.

## 3 Specific issue in metaproteomics based on mass spectrometry with database search

**Fig. 1:**
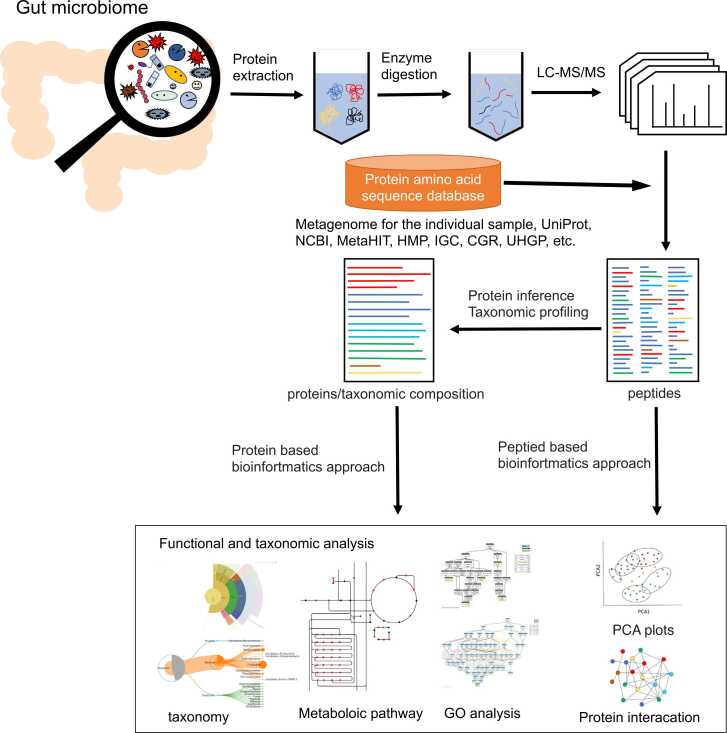
Metaproteomics procedure. Proteins are extracted from fecal samples, digested by enzymes such as trypsin, and analyzed by LC-MS/MS. Peptides are identified by a large-scale database search for the attribution of spectra. Sequences that are not found in the database cannot be identified. Recently, bioinformatics research on peptide base, in which a taxonomy and GO analysis are performed directly on the obtained peptides, has been actively conducted using Unipept, MetaGOmics, and other bioinformatic tools [47], [48]. Further bioinformatics research is being conducted to infer protein and species from peptides. These studies on multiple species are similar but more complex than single-species proteomics.

Fig. 1 shows the flow chart of metaproteomics. The procedure was very similar to that used for proteomics. In shotgun proteomics, proteins prepared from fecal samples are digested with enzymes such as trypsin, and the elution time, MS, and fragmented MS/MS spectra are obtained by liquid chromatography and MS. A database of protein amino acid sequences was used to identify peptide sequences from MS/MS spectra (LC-MS/MS). Protein sequences in the database were also trypsin-digested *in silico*, and peptides with masses very close to MS (precursor) were retrieved and further matched with MS/MS spectra. Peptides were identified with matching scores (peptide spectrum match (PSM)). The obtained PSMs were assembled into proteins using the amino acid sequence database, and the species to which they belonged were also estimated simultaneously. A drawback of proteomics is that the obtained peptides belong to multiple proteins; similarly, in metaproteomics, a problem arises wherein peptides often belong to multiple species. In this review, we do not deal with such problems but discuss the three issues mentioned in the introduction to be solved in the procedure of obtaining PSM from mass spectrometry.

### 3.1 Diversity of organisms in the microbiome leads to enlargement of the database, with respect to issues in database construction

There are over 1000 species of intestinal microbiome, and the number of individual variations is very large. Unlike proteomics, it is not known in advance which species are present in a sample. Therefore, database construction is a major challenge in metaproteomics database searches. The choice of the protein database directly affects the sensitivity of peptide identification. The choice of database may increase the estimated false discovery rate (FDR) (that is, the threshold for accepting a putative protein identification), which can lead to rejection of the PSM (discussed in Section 3.2). For the gut microbiome, database selection is difficult because the bacterial species included in the database are not known in advance. This problem is further complicated by the research community's adherence to historical FDR practices [49], [50].

#### 3.1.1 Methods for database construction in metaproteomics

Blakeley-Ruiz et al. proposed the selection of appropriate databases for metaproteomics and classified existing database construction methods into four categories, as shown in Table 1 [51]. The optimal database should only contain proteins and post-translational modifications that are present in the sample and can be detected by MS [50]. From this viewpoint, the matched metagenome database is the best among the four categories [50], [51], [52]. It is the most compact database with the most accurate answers as it provides the species in the sample that were unknown by prior metagenomic analysis, along with their sequences. However, the main disadvantages of matched metagenomic databases are that they are costly and require large sample volumes, as it is important to obtain high-quality sequences of the genome. Similarly, the unmatched reference database (also called pseudo metagenome [52]) was constructed by identifying the species by 16S rRNA gene analysis, and then merging the sequence databases of the species into the database. At first glance, this appears to be efficient. If the genome information of the detected species is available in the database, it can be used to construct a database that is nearly equivalent to a matched metagenome.

**Table 1:** Four categories of the protein amino acid sequences for metaproteomics.

Category	Description
Matched metagenome database	A database composed of protein amino acid sequences obtained by metagenomic analysis of the samples to be analyzed.
Unmatched metagenome database	A database derived from previous metagenomics and isolate sequencing researches of a specific system such as gut, oral microbiome. Sometimes these metagenomes are published as gene catalogs.
Unrestricted reference database	Sequences obtained from public databases. For example, NCBI's refseq [53] and Uniprot [54]. 80–90 % of the MAGs in the Metagenome Project belong to unnamed species that do not exist in public repositories.
Restricted reference database	A database that collects sequences of necessary organism species based on information on the organism species obtained from 16S rRNA gene analysis of samples to be analyzed.

#### 3.1.2 Unmatched metagenome database based on metagenome analysis

The unmatched metagenome database is specialized for specific microbiomes, such as enterobacteria, and is based on metagenomic analyses of cohorts. Table 2 summarizes the major databases [55], [56], [57], [58], [59], [60], [61], [62], [63]. Considering that articles on human intestinal bacteria have been published since 2009, it is evident that metaproteomics has developed alongside the development of these databases. In the early stages of Metagenomics of the Human Intestinal Tract [55], [56], [58], Human Microbiome Project [57], and Integrated Gene Catalog (IGC) [59], it was important to increase the number of samples to construct the database.

**Table 2:** Protein amino acid sequence database of gut microbiome.

Database name	The number of sequences	Description	Ref.
MetaHIT	3,299,822	Metagenomics of the Human Intestinal Tract (MetaHIT) The metagenome from a sample of 124 European individuals was obtained by mapping 89 genes representative of human intestinal bacteria. EBI accession ERA000116	[55], [56], [58]
	4,267,985	In addition to the sample of 124 European individuals, a metagenomic analysis of a sample from 145 Chinese people was added.	
HMP	5,140,472	Human Microbiome Project (HMP) The HMP database was established by the Human Microbiome Project Consortium supported by The National Institutes of Health (NIH), USA. It was obtained from the analysis of 5298 samples collected from 242 individuals.	[57]
IGC	9,868,476	The Integrated Gene Catalog (IGC) https://db.cngb.org/microbiome/genecatalog/genecatalog_human/	[59]
JPGM	4,854,719	Japanese gut microbiome (JPGM) Metagenomic data of intestinal microbiota from 106 Japanese was collected by sequencing of fecal DNA samples using next-generation sequencing (NGS).	[60]
CGR	5,749,641	Culturable Genome Reference (CGR) A collection of 1520 non-redundant, high-quality draft genomes was generated from more than 6000 bacteria cultured from healthy human fecal samples.	[61]
HBC	5,139,245	Human Gastrointestinal Bacteria Culture Collection (HBC) Samples were cultured and purified from bacteria isolated from fecal samples of 20 adults based in the United Kingdom (n = 8) and North America (n = 12). ENA project numbers ERP105624 and ERP012217.	[62]
UHGP	170,602,708	Unified Human Gastrointestinal Protein (UHGP) A database constructed by reanalyzing almost all publicly available human microbiome. https://www.ebi.ac.uk/metagenomics/genomes	[63]

The database constructed in 2010 by Qin et al. [55] was built as part of the Metagenomics of the Human Intestinal Tract (MetaHIT) project. Fecal specimens were collected from 124 people from Denmark and Spain, including healthy, overweight and obese human adults, as well as patients with IBD. Metagenomic analysis, assembly, and characterization using Illumina Genome Analyzer technology yielded 3.3 million non-redundant microbial genes, which is 150 times greater than the number of genes in the human gene complement. The analysis contained 536,000 potent unique genes corresponding to approximately 1000 bacterial species with average-sized genomes. Le Chatelier et al. performed metagenomic analysis on a Danish sample of 123 non-obese and 169 obese individuals, and obtained comparable numbers of genes. [56]. MetaHIT also performed a metagenomic analysis of 145 Chinese individuals (71 cases and 74 controls) to analyze type 2 diabetes and integrated the first Qin analysis to obtain a catalog of 4.2 million genes for 2890 bacteria species [58].

The National Institutes of Health (NIH)-funded Human Microbiome Project Consortium established a population-wide framework to develop metagenomic analysis protocols [61]. These protocols were rigorously standardized and quality controlled for simultaneous use at four sequencing centers (BCM Human Genome Sequencing Center, the Broad Institute of Massachusetts Institute of Technology (MIT) and Harvard, the J. Craig Venter Institute, and the Genome Institute at Washington University School of Medicine), these protocols were rigorously standardized and quality-controlled for simultaneous use. Metagenomic analysis was then performed using samples obtained 1–3 times from 15 to 18 sites, including the oral cavity and skin of 242 individuals. HMP yielded 5 million genes, which are more than those obtained by MetaHIT, although it should be noted that HMP analyzed a variety of commensal bacteria beyond those in the gut. IGC is the largest database at that point constructed by integrating 1018 known samples and 249 newly analyzed samples in MetaHIT (760 in Europe, 363 in China, and 138 in the United States), and 3499 analyses from prokaryotic genomes [59]. The IGC contains approximately 10 million proteins and is widely used.

#### 3.1.3 Database of high-quality genomes in culture

The Culturable Genome Reference (CGR) [61] and the Human Gastrointestinal Bacteria Culture Collection (HBC) [62] have constructed high-grade genomes from human fecal samples by culture. CGR has obtained 1520 genomes with 5.75 million genes including 264 newly acquired species. HBC has obtained 1354 genomes including 105 novel species with 5.14 million genes. Although the number of sequences in CGR and HBC is approximately 60% of that in the IGC, the genome is characterized by its high grade in culture.

The Unified Human Gastrointestinal Protein (UHGP) database [63] is currently the largest database with 1.7 billion protein amino acid sequences. The UHGP has collected and analyzed almost all genes currently in the publicly available genome to build a comprehensive microbiome sequence database. Although it is possible to construct a large database, may not include all genes, as the distribution of intestinal flora differs greatly among individuals. This issue will continue to be addressed when using databases with unmatched metagenomes.

#### 3.1.4 Differences in the composition of the bacterial flora in different regions

Efforts to increase the number of samples collected and to elucidate a large number of genomes continue, including those in culture. Nevertheless, as mentioned above, the distribution of intestinal microbiota varies greatly from person to person. The Japanese gut microbiome [60] is a database of the intestinal microbiota of the Japanese population that contains nearly 5 million genes (proteins). Clustering of genes with IGC, which is mainly from Europe, the United States, and China, revealed that only approximately 40 % of the genes were common to each other. However, when KEGG Orthology [64], a functional hierarchy of genes in the KEGG database, was used to annotate the functions of the genes, 90 % were identified as common. This is not ideal from the perspective of proteomics, as the sequence must first be identified to elucidate its function. This problem must be solved for metaproteomics to use the database by unmatched metagenome. The choice is between costly high-quality metagenomic analyses and large-scale unmatched metagenome databases.

#### 3.1.5 Comparison between database categories

The metaproteomics analysis of leukemia patients with MRE [4] in Section 2.6 is interesting in that it discusses the identification of PSM using various databases in conjunction with clinical metaproteomic studies. Here, we show metaproteomics with two databases, the matched metagenome database and IGC, as summarized in Table 3. The number of sequences in the sample-specific metagenomic database was approximately half that in the IGC. A little less than half of these (17 million) are shared with IGC. Although the number of sequences was approximately half, the number of identified peptides was around 20 % greater than that of the IGC. The number of commonly identified peptides in the database was 170,000. The processing time of the search is also discussed. With the IGC database, it took 1834 h to search 212 samples using 10 cores on a Xeon Gold 6150 CPU at 2.7 GHz, and the output data was 1.78 TB. However, using the specific metagenome database, processing was completed in only 310 h using just two cores, and the amount of data was 830 GB.

**Table 3:** Database, the number of sequences used, and identified peptides in metaproteomics for Leukemia patients with multidrug-resistant Enterobacteriaceae (MRE) (in Ref. [4]).

Database	Category	The number of sequences used	Identified peptides
Sample specific metagenome	Matched metagenome	41,081,954	255,442
Integrated Gene Catalog	Unmatched metagenome	81,680,329	208,495

### 3.2 Various depressions caused by large databases, with respect to issues in false identification in the database search

**Fig. 2:**
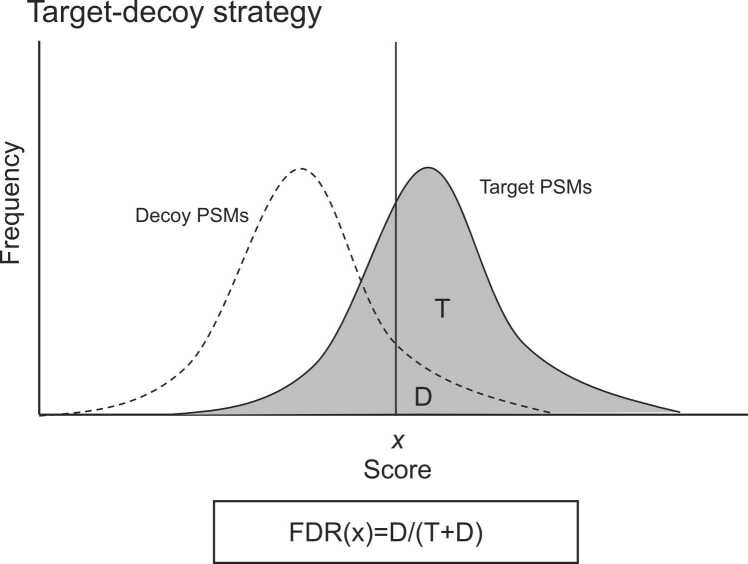
Distribution of target peptide spectrum matches (PSMs) and decoy PSMs in target-decoy search. ‘x′ at horizontal axis means score threshold for a certain false discovery rate (FDR).

PSM was identified based on the score obtained for the degree of agreement between the spectrum and peptide. Therefore, a certain percentage of misidentifications occurs when numerous PSMs are obtained. A target-decoy search is used in current peptide identification from MS/MS to control false identifications [65]. In this method, a false database (decoy) is prepared for a protein amino acid sequence (target), and the ratio of false identifications (false discovery rate (FDR)) is calculated as the ratio of decoy PSMs obtained from the false database to the total PSMs at a certain score. Fig. 2 shows the distribution of the target and decoy PSMs plotted against the PSM matching score. When the score x is set as the threshold value, x is determined such that the area of D (below the dotted line) is a certain percentage (*e.g.*, 1 %) of T (above the dotted line and below the distribution of practice), and the target PSM is accepted as the identified peptide. For the decoy database, it is necessary to construct a sequence database that is equivalent to the target database in terms of amino acid composition and sequence length distribution but cannot be identified as the target. Elias and Gygi proposed the following five guidelines [65]:“

1. Similar amino acid distributions as target protein sequences,
2. Similar protein length distribution as target protein sequence list,
3. Similar numbers of proteins as target protein list,
4. Similar numbers of predicted peptides as target protein list,
5. No predicted peptides in common between target and decoy sequence lists. ”.

In general, to simply satisfy these five guidelines, decoy databases are often created by reversing the N- and C-termini of the target amino acid sequence. This method of creating a decoy database is called the reverse method. There is also the shuffle method, which replaces amino acids in an amino acid sequence, and the “random” method, which generates an amino acid sequence using random numbers in a way that preserves the distribution of amino acids and length distribution. In some cases, a combination of these methods is used to create the product [66].

#### 3.2.1 Issues in false identification in database searches

**Fig. 3:**
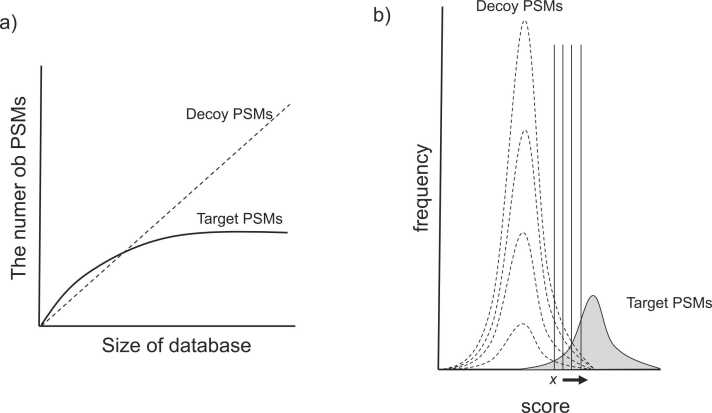
Size dependency of the target and decoy database and behavior of FDR score thresholds. a) Size dependency of target and decoy databases. b) Behavior of FDR score thresholds depending on the size of the decoy database.

The relationship between the database size and the number of PSMs (Fig. 3a) shows that the number of PSMs stops increasing at a certain point because the target PSM saturates when all peptides in the sample are identified. On the other hand, decoy PSMs are randomly identified because they happen to be hit by chance and thus increase in proportion to the size of the database. Fig. 3b shows how the distribution of decoy PSMs changed as the database size increased. The score distribution was assumed to expand. The ratio of decoy PSMs to target PSMs in the high-score region increases as the number of decoy PSMs increases because the tail of the decoy PSM distribution extends to the high-score region. This moves the score threshold, x, to the high-score region. This indicates a decrease in PSM, suggesting that the way the database is constructed affects peptide identification. This is a major problem for current proteomics using large databases such as metaproteomics. Currently, it is necessary to construct a database that contains only the minimum necessary sequences as much as possible, similar to the best database described in Section 3.1. Various studies have been conducted on database construction and identification methods. However, in the current situation, metagenomic analysis of measured samples is a good choice to construct a protein amino acid sequence database in advance. Even in such cases, database construction based on high-quality metagenome analysis is very effective. However, care must be taken when constructing incomplete amino acid sequences from incomplete genomes. As metaproteomics of enterobacteria uses a large database, it can be easily inferred that the number of randomly hit decoy PSMs greatly exceeds the target PSM. This is thought to be the reason for the reduced sensitivity of metaproteomics. We also note that Guideline 4 of Elias and Gygi is broken.

#### 3.2.2 Attempt to solve the FDR problem by narrowing down the database using the multi-step method

There are several approaches to address the problem of reduced identified peptides in target-decoy searches using large-scale databases. These can be broadly divided into two types, step-by-step narrowing searches [67], [68], [69], [70], [71] and post-processing approaches such as rescoring [72], [73], [74], [75]. The former is an attempt to solve the problem of target-decoy search by selecting important sequences by repeating the database search.

The two-step method proposed by Jagtap et al. [67] has been widely used. In the first step, only a target search is performed, and a subset of proteins containing the obtained PSM is constructed without considering the FDR. In the second step, a target-decoy search with FDR control is performed. Jagtap et al. performed a metaproteomic analysis of oral bacteria and found that the number of identified host (human) peptides increased by 125 % while the number of identified microbial peptides doubled using the two-step method compared to the usual target-decoy search. The number of identified PSMs increased with repeated refinement. Because only those identified in the target database are selected in the first step, it is possible that the decoy database search space is substantially smaller than the actual database size in the second step. Specifically, target PSMs with higher scores are expected to be identified exclusively as target PSMs, which are already identified in the first step, and there is a concern that the number of identified decoy PSMs may be limited. It should be noted that the apparent increase in PSM identified may be due to the underestimation of decoy PSM, as discussed in [69].

Kumar et al. proposed a method called sectioning which divides a database into several sections [69]. Sequences containing PSMs that satisfied the target FDRs were selected from each of the divided databases and were merged with an equal number of randomly selected sequences which functioned as decoys. This integrated database was used for the final database search. The study used the Simplified Human Intestinal Microbiota (SIHUMI) dataset for validation [76]. The SIHUMI datasets are composed of mass spectrometric data (denoted as SIHUMI.raw) and 29,557 protein amino acid sequences (denoted as SIHUMI.fasta) from eight species: *Anaerostipes caccae*, *Bacteroides thetaiotaomicron*, *Bifidobacterium longum*, *Blautia producta*, *Clostridium butyricum*, *Clostridium ramosum*, *Escherichia coli*, and *Lactobacillus plantarum*. The analysis of SIHUMI.raw using SIHUMI.fasta yielded 50,651 PSMs (FDR < 0.01). They constructed a model database by adding 1,442,786 archaeal proteome sequences from the NCBI-NR database as entrapment sequences in SIHUMI.fasta including contaminant sequences. Although the conventional target-decoy search reduced the PSM by more than 10 %, the 2-step method of Jagstep et al. yielded 50,246 PSM, and the sectioning method with 30 segments yielded 47,232 PSM. The false identification rate of PSMs identified as archaeal proteins identification in the model database analysis was evaluated and was 1 % for the conventional target-decoy method defined by the FDR, but 11 % for the two-step method. The 30-segment sectioning method was able to suppress the false identification rate to approximately 1.5 % using the 1 % FDR condition. Although the number of PSMs obtained by the sectioning method was smaller than that by the two-step method, the false identification rate was lesser than that by the two-step method in terms of FDR control.

#### 3.2.3 Attempt to solve FDR problem by post-scoring methods

In Section 3.2.2, we introduced a method to obtain a large number of PSMs by efficiently constructing a subset of the sequence database. Here, we introduce a method for solving the problems of false positives and false negatives by re-evaluating the scores obtained as a result of a conventional target-decoy search. Post-scoring includes methods such as percolator [72], peptide prophet [73], Tool-Independent and Data-Dependent PSM rescoring (TIDD) [74], and BH-FDR (Benjamini-Hochberg framework) [75]. Percolator uses the matching score as a result of the conventional target-decoy search (*e.g.*, XCorr in SEQUEST [77]), *m/z*, ion valence, and LC retention time as inputs for a support vector machine (SVM), thereby controlling the FDR with scoring optimization. The peptide prophet [73] was developed as a method to classify targets and decoys using only target PSMs by rescoring, noting that the distribution of low-scoring target PSMs is similar to that of decoy PSMs. In general, post-scoring methods improve peptide identification rates by applying machine learning techniques and by re-scoring with a combination of scores from database search engines and properties, such as elution time and delta *m/z*. The data used for machine learning depend on and must be optimized for each search engine. The TIDD proposed by Li et al. [74] uses SVM to optimize scores similar to the percolator but introduces parameters that are independent of search engines. This makes it possible to reliably obtain PSMs regardless of the search engine used. Couté et al. [75] proposed a post-processing approach to directly control FDR. A significant reduction in the decoy PSM was achieved by correcting the p-value of peptides identified by MS/MS for multiple testing using BH-FDR [78]. It is believed that these studies are helping to gradually solve the FDR problem. It is also noteworthy that decoy-free methods such as peptide prophet and BH-FDR have been developed.

### 3.3 Improvement of spectral acquisition methods in mass spectrometry, with respect to issues in spectrum acquisition

Metaproteomics is likely to contain a large variety of peptides, owing to the diversity of bacterial species in a sample. A common approach in metaproteomics is data-dependent acquisition (DDA) mass spectrometry, in which peptides are identified in the order of abundance. However, the performance of DDA has been reported to decrease with increasing sample complexity [79]. Data independent acquisition (DIA), which has become widely used with the establishment of the sequential windowed acquisition of all theoretical fragment ion mass spectra method (SWATH-MS), can acquire more spectra than DDA [80], [81]. The advantage of the SWATH-MS method is that it can quantify multiple molecules simultaneously, enabling comprehensive protein quantification based on all MS/MS spectral data obtained from a sample in a single measurement. DIA methods have recently been applied in metaproteomics [82], [83], [84].

To study the involvement of gut bacteria in the pathogenesis of colorectal cancer (CRC), Long et al. performed a cohort analysis on the gut microbiome of patients with CRC (N = 14) and healthy volunteers (N = 14) using DIA label-free quantitative proteomics [82]. Using the DIA proteomics strategy, 30,062 proteins were identified from gut bacteria present in the healthy cohort and CRC patients. This was higher than the results of a recently published metaproteomics from fecal samples. The gut microbiota of CRC patients and healthy control volunteers was characterized. They found the abundance of 341 microbial proteins was significantly different in CRC patients compared to the healthy population. In addition, several new taxonomic abundance variations were suggested in CRC patients. Future development of clinical diagnostics is anticipated.

Application of DIA in metaproteomics began only recently, and tools to analyze the spectra obtained from complex samples are lacking. Aakko et al. developed diatools, an approach which uses DDA measurements to construct a peptide library for the identification of DIA spectra [83]. Pietilä et al. developed glaDIAtor, which analyzes DIA directly without library construction using DDA [84]. While the former DDA-assisted analysis method can identify many peptides when analyzing a mixed sample of 12 different bacteria, the latter approach can identify more peptides when analyzing complex samples such as feces. The advantage of the latter approach is that the DDA measurement can be omitted, and complex spectra can be analyzed without DDA for limited samples, such as in actual clinical proteomics.

### 3.4 New challenges due to diversity and larger databases

In the discussion so far, we have seen that FDR control is a hindrance to obtaining PSM in analyzes using large databases, such as those in metaproteomics. This is also the outcome of the target decoy search. However, even if FDR is well controlled, the reliability of PSM with low scores is considered low. For example, for a peptide of eight residues, 14 b- or y-ions are generally detected by MS/MS, but in some cases, only a few ions are detected, and the peptide is identified as a PSM. In such cases, a score threshold may be set to exclude unreliable PSMs. In metaproteomics, many organisms have similar sequences because of the large number of closely related species in their microbiome. Naturally, many digested peptides have high degrees of identity. Therefore, if the amino acid sequence of the protein of the original species is not in the database, it is possible that a matching PSM will appear. Therefore, there is great concern regarding false identifications using different approaches than with FDR control. It is expected that these problems will be solved by the recent development of the "decoy free" method in terms of improving the reliability of PSM. A *de novo* identification method without a database search has also been studied [85], [86], [87]. *De novo* identification is used to determine the amino acid sequence directly from the *m/z* difference of fragments in the MS/MS spectrum. Therefore, although *de novo* identification is reliable, it is not possible unless a significant number of ions is observed in the MS/MS spectra. This has the disadvantage that PSM is inevitably reduced. In recent years, studies have been conducted to combine *de novo* identification with deep learning to obtain more PSMs [86]. The method of choice differs depending on whether you want to know the general function or trend of the species, even if there are many false identifications, or if you want to examine the detailed function of the microbiome. In this regard, there are always situations in which a small number of highly reliable identifications using *de novo* methods are important.

## 4 Summary and outlook

In this review, we discussed the usefulness of metaproteomic analysis of intestinal bacteria for the elucidation of host-microbiome communication in disease and the current issues in analysis by mass spectrometry to obtain PSM. As dysbiosis of intestinal bacteria has been recently identified as a possible cause or concomitant of various diseases, metaproteomics is expected to become as routinely used as proteomics as a powerful tool for clarifying both species and functions. To this end, it is important to overcome the issues of database construction, FDR control in the analysis using database search, development of software tools for DIA acquisition of mass spectrometry, and to develop novel analytical methods to obtain reliable PSM, such as *de novo* search. As the development of decoy-free methods is underway, the emergence of more convenient and reliable methods than target-decoy search will greatly advance the field of metaproteomics.

## CRediT authorship contribution statement

**Nobuaki Miura:** Investigation, Methodology, Writing – original draft. **Shujiro Okuda:** Supervision, Writing – review & editing, Funding acquisition, Project administration.

## Conflicts of Interest

The authors have no conflicts of interest.
